# Effects of dietary fucoidan and exercise on the gut microbiome, fecal metabolome, and tumor-related responses in a murine colorectal cancer model

**DOI:** 10.3389/fcimb.2026.1926528

**Published:** 2026-09-18

**Authors:** Lin Zhong, Bhagyalakshmi Purushothaman, Xingxiang Wang, Yucheng Zhang, Xiangze Sun, Jiyi Jiang, Karamveer Karamveer, Youming Zhang, Qiang Tu, Boopathi Seenivasan

**Affiliations:** 1 Institute of Synthetic Biology Industry, Hunan University of Arts and Science, Changde, Hunan, China; 2 Helmholtz International Lab for Anti-Infectives, Shandong University-Helmholtz Institute of Biotechnology, State Key Laboratory of Microbial Technology, Shandong University, Qingdao, Shandong, China; 3 College of Life and Geographic Sciences, Key Laboratory of Biological Resources and Ecology of Pamirs Plateauin Xinjiang Uygur Autonomous Region, Kashi University, Kashi, China; 4 School of Marine Science and Engineering, Qingdao Agricultural University, Qingdao, China; 5 Department of Pediatrics, Penn State College of Medicine, Hershey, PA, United States; 6 Yuelushan Laboratory, Changsha, Hunan, China

**Keywords:** colorectal cancer, dietary fucoidan, gut microbiome, host–microbiome interaction, metabolomics

## Abstract

Dietary fucoidan, a sulfated polysaccharide derived from brown seaweed, has attracted increasing interest for its potential to influence gut microbial and metabolic responses. However, the individual and combined effects of dietary fucoidan and exercise on host–microbiome interactions during colorectal cancer development remain incompletely understood. In this preventive study, mice received fucoidan at 300 mg/kg (F1) or 600 mg/kg (F2), alone or in combination with low-intensity exercise at 12 m/min for 30 min or moderate-intensity exercise at 15 m/min for 40 min, for six weeks before MC38 tumor induction. The interventions were associated with distinct changes in gut microbial composition and fecal metabolic profiles. Notably, the X2F1 group showed enrichment of *Lactobacillus murinus* together with alterations in bacterial taxa linked to short-chain fatty acid metabolism. Metabolomic analysis further revealed changes in amino acid, bile acid, and central carbon metabolism, indicating coordinated microbial and metabolic remodeling. The X2F1 group also showed a marked reduction in tumor burden, while low-dose fucoidan alone produced a clear tumor-associated response and broad changes across microbial and metabolic outcomes. Tumor gene-expression analysis showed increased p53 and Bax expression and reduced Bcl-2 and Ki67 expression in several intervention groups, consistent with apoptosis- and proliferation-related molecular responses. These findings suggest that dietary fucoidan, alone or in combination with exercise, is associated with coordinated changes in the gut microbiome, fecal metabolome, tumor burden, and tumor-related molecular profiles in an MC38 colorectal cancer model.

## 1 Introduction

Dietary polysaccharides derived from marine sources are increasingly recognized as functional components that can influence host metabolism through interactions with the gut microbiome. Among these, fucoidan, a sulfated polysaccharide rich in fucose and sulfate groups along with minor sugars such as galactose, mannose, and uronic acids, has attracted considerable attention due to its structural complexity and wide range of biological activities (Praveen et al., 2019). Importantly, its structural features, including the degree of sulfation and molecular weight, are closely linked to its biological function, particularly its ability to interact with gut microbes (Wang et al., 2019). As a nondigestible carbohydrate, fucoidan escapes digestion in the upper gastrointestinal tract and reaches the colon, where it becomes available for microbial fermentation, thereby influencing both microbial composition and metabolic output (Zheng et al., 2023; Li et al., 2024).

The gut microbiota acts as a central metabolic interface between diet and host physiology. It contributes to nutrient processing, generates bioactive metabolites, and plays a key role in maintaining immune balance and intestinal barrier function (Velagapudi et al., 2010; Nicholson et al., 2012; Jakobsson et al., 2015). Disruptions in this system have been strongly linked to colorectal cancer (CRC), where shifts in microbial composition and metabolite profiles can promote inflammation, impair epithelial integrity, and influence tumor-related signaling pathways (Louis et al., 2014). In particular, metabolites such as SCFA, bile acids, and tryptophan-derived compounds are now recognized as important mediators that connect microbial activity with host metabolic and immune responses (Zhang et al., 2019).

Recent studies suggest that fucoidan can reshape the gut microbiota and alter its metabolic activity, often leading to enrichment of beneficial taxa and increased production of metabolites associated with intestinal and systemic health (Li et al., 2024). These microbiome-mediated effects highlight its potential as a functional food ingredient capable of influencing disease-related metabolic pathways, including those involved in cancer. Alongside dietary components, physical activity is another important factor that can modulate the gut microbiome. However, its effects are not uniform and depend on factors such as exercise intensity, duration, and host physiology (Lamoureux et al., 2017; Yang et al., 2021; Suryani et al., 2022). Moderate exercise is generally associated with beneficial microbial and metabolic adaptations, whereas excessive or prolonged high-intensity exercise may disrupt gut homeostasis and barrier function (Khan et al., 2020; Clauss et al., 2021).

Importantly, clinical and preclinical studies have provided evidence for the independent benefits of fucoidan and exercise in CRC. In patients with CRC, fucoidan supplementation has been associated with improved disease control and reduced treatment-related fatigue (Ikeguchi et al., 2011; Tsai et al., 2017, 2023). Similarly, clinical trials have shown that exercise can improve survival outcomes, physical fitness, and cancer-related fatigue in CRC patients (Singh et al., 2020; Zhang et al., 2025). However, clinical evidence on the combined use of fucoidan and exercise in CRC is still lacking. Both interventions have been linked to changes in the gut microbiota, inflammatory responses, and immune function, but whether their combined use can produce greater anticancer effects than either intervention alone remains unclear.

In this study, we used a preventive intervention design to investigate whether six weeks of dietary fucoidan supplementation, alone or in combination with controlled exercise, could influence subsequent colorectal tumor development in an MC38 murine model. Fucoidan was administered at 300 or 600 mg/kg, either alone or together with low- or moderate-intensity exercise, before tumor induction. We evaluated the effects of these interventions on gut microbiota composition, predicted microbial functions, fecal metabolomic profiles, tumor development, and tumor-related molecular responses. We hypothesized that prior dietary fucoidan exposure, particularly when combined with exercise, would be associated with selective changes in functionally relevant microbial taxa and coordinated alterations in microbiota-related metabolites, rather than changes in microbial diversity alone. By integrating microbiome, metabolome, tumor burden, and apoptosis- and proliferation-related gene-expression data, this study aimed to determine how fucoidan, alone and in combination with exercise, is associated with changes in the gut–microbiome–metabolism–tumor axis during colorectal cancer development.

## 2 Materials and methods

### 2.1 Animals

Six-week-old male C57BL/6 mice were obtained from Ji’nan Pengyue laboratory Animal Breeding Co., Ltd., China. The animals were housed in a typical laboratory setting with a 12-hour light/dark cycle and maintained at 22 °C and 60% relative humidity. All mice were given sterilized drinking water and fed an identical batch of standard laboratory chow to reduce potential environmental variability. To ensure adequate acclimatization throughout the trial, all mice were handled daily for at least a week prior to the start of the studies. Following acclimatization, mice were randomly allocated to the experimental groups using random number sequence. Cage positions were also randomized within the housing room to minimize potential cage-related effects. Sample sizes of N = 5 per group for tumor-related outcomes and N = 3 per group for microbiome and metabolomics analyses were chosen based on commonly used group sizes in comparable preclinical oncology and microbiome studies, while also considering the ethical need to minimize animal use. The Laboratory Animal Ethics Review Committee of Shandong University approved every experimental procedure (SYDWLL-2025-144). All animal experiments were conducted in accordance with the ARRIVE guidelines and the EU Directive 2010/63/EU for the protection of animals used for scientific purposes.

### 2.2 Experimental design

A total of 50 male mice (n=5 per group) were randomly divided into ten groups: Con (control), BFEXO (baseline group with no exercise or fucoidan before treatment), F1 (300 mg/kg body weight (b.w) per day fucoidan), F2 (600 mg/kg b.w per day fucoidan), X1 (low intensity exercise), X2 (moderate intensity exercise), and four combined treatment groups including X1F1, X1F2, X2F1, and X2F2 that received both exercise and fucoidan at the corresponding doses. Our group size was consistent with previous MC38 syngeneic tumor studies and was considered sufficient to evaluate treatment-related differences while minimizing animal use in accordance with ethical principles (Selby et al., 2016). Fucoidan (CAS No. 9072-19-9, ≥95% purity) was dissolved in sterile deionized water, and administered daily by oral gavage. The concentrations of the fucoidan was chosen based on the previous studies to provide two biologically relevant oral doses within a translationally meaningful range (Li et al., 2005). Body-surface-area conversion of these doses corresponds to approximately 1.46 and 2.92 g/day for a 60-kg adult, respectively, which falls within the range of fucoidan doses previously evaluated in human studies (Sakai et al., 2019; Cousins et al., 2025). In cancer populations specifically, fucoidan has been administered at 4.0 g/day in advanced cancer patients (Tsai et al., 2017). These comparisons suggest that the mouse doses used in this study fall within a range that is relevant to human fucoidan supplementation. The X1F1, and X2F1 groups received a low dose of 300 mg/kg b.w per day, whereas the X1F2, and X2F2 groups received a high dose of 600 mg/kg b.w per day (**Figure 1**).

**Figure 1:**
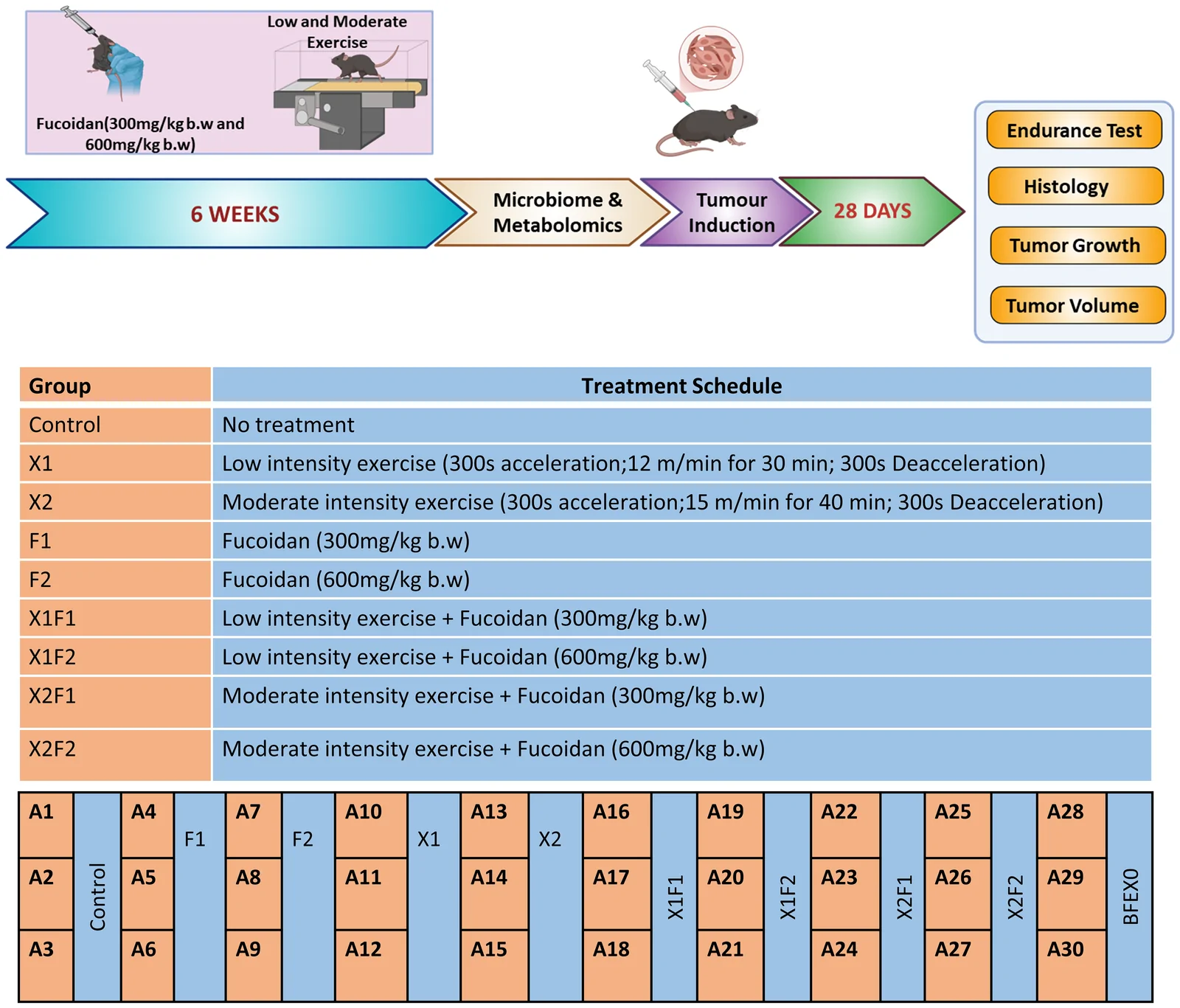
Schematic representation of the experimental design illustrating exercise and fucoidan interventions along with Sample coding scheme for all groups included in the gut microbiome analysis.

All mice were acclimated with treadmill use for five days before the training program. During the acclimatization phase, mice were subjected to 5 m/min for 10 to 20 minutes to assist them adapt to the equipment. Following acclimatization, a six-week forced running program was carried out five days a week on six-lane treadmill fitted with a rest platform and an electronic control unit (Beijing Dingguo Changsheng Biotechnology Co., Ltd., China). Mice in the X1, X1F1, and X1F2 groups underwent a low intensity exercise training that began with a 300 second acceleration warm up at 12 m/min, followed by 30 minutes of running at the same speed, and ended with a 300 second deceleration cool down at 8 m/min. In contrast, the X2, X2F1, and X2F2 groups completed a moderate intensity protocol that started with a 300 second acceleration warm up at 15 m/min, continued with 40 minutes of running at 15 m/min, and concluded with a 300 second deceleration cool down at 8 m/min (Lamoureux et al., 2017). The treadmill protocol was designed to represent submaximal aerobic exercise rather than exhaustive training. This approach reflects exercise programs commonly used in humans, where moderate continuous exercise is typically performed for 30–50 minutes at low-to-moderate intensity, with structured warm-up and cool-down periods (Dourida et al., 2019; Nindorera et al., 2025). For both procedures, an electric stimulus of 0.8 mA was applied to stimulate running as needed. After each session, the treadmill belt was wiped with 70 percent ethanol, rinsed with sterile water, and fully dried before the next group was placed on the equipment. Sedentary mice were subjected to the same experimental conditions, including handling, noise, vibration, and brief food and water restrictions, but they did not exercise. If a mouse paused during a run, it was gently pushed from the rest platform back onto the treadmill to resume locomotion. The overall condition of the mice, including their behavior, glossiness of hair, and responsiveness to stimuli, was monitored daily. Food consumption was tracked by weighing and giving measured amounts of chow to each cage every three days. Uneaten food was collected and weighed to determine individual consumption. Body weight was measured every week to monitor changes in body composition. The faeces of mice were sampled within 24 h of the last intervention. The mice were put into a clean cage covered with sterile filter paper in the ventilation cupboard. Fecal samples were collected immediately after the mice were gently stimulated to defecate with a brush, and faeces were collected for each mouse and used for 16S rRNA microbiome and metabolomics study.

### 2.3 Gut microbiota analysis

Microbial DNA was extracted and subjected to 16S rRNA sequencing to assess gut microbiota composition and diversity across the treatment groups. For gut microbiome analyses, n=3 biological replicates per group were used, consistent with pilot-scale microbiome studies. This was considered sufficient to capture major treatment-related changes, as technical variation in 16S rRNA sequencing is generally small relative to biological differences between experimental groups, while also minimizing animal use and sequencing burden (McAdams, 2024). Genomic DNA was extracted from mouse feces samples using the CretMag™ Power Soil DNA Kit (CretBiotech, China), following the manufacturer’s protocol. The quality of the extracted DNA was assessed by 1% agarose gel electrophoresis, and its concentration and purity were measured using a NanoDrop 2000 UV-vis spectrophotometer (Thermo Scientific, Wilmington, USA). To analyze bacterial composition, the V3–V4 hypervariable regions of the 16S rRNA gene were amplified using the primer pair 341F (5’-CCTAYGGGRBGCASCAG-3’) and 806R (5’-GGACTACHVGGGTWTCTAAT-3’) on an A200 PCR thermocycler (LongGene, China). All reactions were performed in triplicate to ensure reproducibility. Amplified products were separated on a 2% agarose gel and purified using the AxyPrep DNA Gel Extraction Kit (Axygen Biosciences, Union City, CA, USA) as per the manufacturer’s instructions. Final DNA concentrations were determined using a Quantus™ Fluorometer (Promega, USA). Purified amplicons were combined in equal concentrations and subjected to sequencing on an Oxford Nanopore Technologies platform at Benagen (Wuhan, China) following standard procedures.

### 2.4 Microbiome analysis

Taxonomic assignment for each ASV was performed using the Silva 138 SSU database in QIIME2 version 2021.4 (Bolyen et al., 2019). Venn diagrams were generated in R version 4.2.2 (R Core Team) to visualize shared and unique ASVs among groups. Alpha diversity metrics, including species richness (ACE, Chao1) and diversity/evenness indices (Shannon, Simpson), were calculated using the vegan package (version 2.6–4) and visualized with ggplot2 (version 3.4.2) to examine shared and unique taxa, and explored overall microbial composition patterns to determine how clearly the groups separated from one another. For beta diversity, Bray–Curtis dissimilarity and Jaccard similarity matrices were computed with the microeco package (version 0.14.1) (Liu et al., 2021) and visualized through NMDS plots generated in ggplot2. Group differences were evaluated using one-way ANOSIM. To identify taxa that significantly distinguished the groups, LEfSe analysis was performed. Heatmaps based on absolute microbial abundance were created using pheatmap (version 1.0.12). Functional predictions were obtained with PICRUSt2 (Douglas et al., 2020), while FAPROTAX was used to predict ecological functional groups. Differences in predicted pathway abundances between groups were assessed using Welch’s t-test followed by Benjamin–Hochberg FDR correction

### 2.5 Metabolomics analysis

For fecal metabolomics, n=3 biological replicates per group were analyzed using a targeted LC–MS/MS metabolomics approach to characterize treatment-associated metabolic changes. The targeted panel included around 300 analytes covering metabolites related to microbial fermentation, amino acid and tryptophan metabolism, bile acid metabolism, central carbon metabolism, nucleotide and cofactor metabolism, lipid metabolism, and microbial-derived aromatic metabolism. Approximately 25 mg of fecal sample was extracted with 700 μL methanol/water (4:1) containing 1 μg/mL L-2-chlorophenylalanine as an internal standard. Samples were homogenized with 5mm beads and sonicated at 35 Hz for 4 min for three cycles. The extracts were then incubated at −40 °C for 1 h and centrifuged, and the resulting supernatants were collected for LC–MS/MS analysis. Equal volumes of all samples were pooled to prepare a quality control sample, which was analyzed alongside the study samples to monitor analytical stability and batch variation. Metabolite analysis was performed by Shanghai Biotree Biotech Co., Ltd. using an Agilent 1290 UPLC system coupled with a SCIEX 6500 QTRAP triple quadrupole mass spectrometer. Chromatographic separation was performed on an ACQUITY UPLC BEH C18 column (1.7 μm, 2.1 × 150 mm) using 5 mM ammonium acetate in water as mobile phase A and acetonitrile as mobile phase B. The gradient was 1% B from 0 to 1.0 min, increased from 1% to 99% B from 1.0 to 8.0 min, maintained at 99% B until 10.0 min, returned to 1% B from 10.0 to 10.1 min, and maintained at 1% B until 12.0 min. The autosampler temperature was maintained at 6 °C and the injection volume was 1 μL. Mass spectrometric detection was performed in multiple reaction monitoring mode using electrospray ionization. The source parameters were set as follows. Curtain gas was 35 psi, ion spray voltage was −4500 V in negative mode, source temperature was 450 °C, and ion source gases 1 and 2 were each set to 50 psi. Data acquisition and quantitative analysis were performed using SCIEX Analyst Workstation software version 1.7.32 and BIOTREE BioBud version 2.0.3. Individual authentic standards were used for absolute quantification of the analyzed metabolites.

### 2.6 Microbiome-metabolome correlation analysis

Genus-level microbial abundances and metabolite profiles were combined with sample metadata and analyzed separately for each experimental group. Metabolites were annotated using KEGG compound identifiers and grouped into superpathways based on KEGG classifications (Kanehisa et al., 2023). We calculated Spearman rank correlations between all microbial genera and metabolite features, after first removing any features that showed no variation (Wickham et al., 2019). For visualization, we selected the top 20 microbial genera with the most variable correlation patterns, along with the top 20 metabolite superpathways showing the highest overall abundance. Correlations at the individual metabolite level were then summarized at the superpathway level by averaging the correlation values of all metabolites within each pathway. Phylum-level taxonomic information was obtained from the NCBI taxonomy database using the taxize package (Chamberlain and Szöcs, 2013). Metabolite superfamily classifications were assigned based on the most common class within each superpathway. Correlation matrices were visualized as hierarchically clustered heatmaps, with phylum and superfamily annotations added for context. These were generated using the pheatmap (Kolde, 2019) and colorspace packages (Zeileis et al., 2020), with a diverging color scale where red indicates positive correlations and blue indicates negative correlations.

### 2.7 Tumor induction

MC-38 cells were grown in RPMI-1640 medium (Gibco) supplemented with 10% FBS and 1% penicillin–streptomycin. After 6 weeks of intervention, MC38 colorectal cancer cells (1 × 10^6^ cells/mouse) were subcutaneously injected into the right flank of each mouse and allowed to grow for next 28 days to check the protective efficacy of gut microbial diversity and their metabolites. Tumor size was measured every 3 days post-injection using a digital caliper. Tumor volume was calculated using the formula: V = (length × width²)/2. At the end of the study, mice were sedated with isoflurane and euthanized via cervical dislocation. From the excised tumor, volume and weight were measured.

#### 2.7.1 Endurance test

To assess how tumor burden affected fatigability, mice underwent a run-to-fatigue test before euthanasia. The protocol consisted of a 600-second acceleration phase reaching 20 m/min, followed by running at 20 m/min until the mice reached fatigue. Fatigue was defined as the point at which a mouse could no longer continue running, even after gentle hand prodding for at least one minute. The total distance covered and the number of electric stimuli received were recorded.

#### 2.7.2 Gene expression analysis

Total RNA was isolated from tumor tissues using the SteadyPure Universal RNA Extraction Kit (Accurate Biotechnology Co., Ltd., Hunan, China) following the recommended protocol. Prior to extraction, tissues were mechanically disrupted using a T Grinder homogenizer (Tiangen Biotech Co., Ltd., Beijing, China) to ensure complete lysis. The purity and concentration of the extracted RNA were evaluated spectrophotometrically (NanoDrop 2000c, Thermo Fisher Scientific, USA), and only samples with acceptable purity ratios (A260/A280 between 1.8 and 2.1) were used for downstream analysis. First-strand cDNA was synthesized from purified RNA using the Evo M-MLV RT Mix Kit with genomic DNA removal (Accurate Biotechnology Co., Ltd., Hunan, China). Quantitative real-time PCR was carried out on a Applied Biosystems real-time PCR system (Thermo Fisher Scientific, USA) using SYBR Green chemistry (SYBR Green Premix Pro Taq Hs, Accurate Biotechnology Co., Ltd., Hunan, China). Gene expression analysis focused on markers associated with apoptosis and tumor progression, including p53, Bax, Bcl-2, VEGF, and Ki-67. GAPDH was used as an internal control and relative transcriptional changes were calculated. Primer details are provided in **Supplementary Table 4**.

#### 2.7.3 Histopathological analysis

The intestinal tissues were carefully excised from the mice and immediately fixed in 4% formaldehyde solution. Following fixation, samples were processed through a graded ethanol series for dehydration and subsequently embedded in paraffin. Tissue blocks were sectioned into approximately 3 µm thick slices using a microtome. The sections were then stained with hematoxylin and eosin (H&E) (Sigma-Aldrich, USA) to evaluate tissue morphology. Stained sections were visualized and imaged using a brightfield microscope. Histological features were further examined and analyzed using SlideViewer software (version 2.6.0.166179).

### 2.8 Statistical analysis

The **Supplementary Table 1** provides a detailed presentation of the various software tools used to analyze the microbiome data. The mean ± standard deviation (SD) is used to display statistical data. GraphPad Prism version 9.0 was used for all statistical analyses. Multiple comparison *post-hoc* analysis was used along with one-way analysis of variance (ANOVA). P < 0.05 was considered statistically significant. For normally distributed data involving multiple groups, one-way ANOVA followed by Dunnett’s multiple-comparisons test was used to compare each intervention group with the corresponding control. Unless otherwise indicated, no direct statistical comparisons were made among intervention groups.

## 3 Results

### 3.1 Gut microbiome analysis

#### 3.1.1 Genomic analysis

After sequencing of 30 samples, a total of 2,595,492 full-length sequences were obtained. Each sample generated at least 44,794 full-length sequences, with an average of 86,516 full-length sequences. The raw sequencing data were processed through barcode demultiplexing, low-quality read filtering, and primer identification and trimming to obtain high-quality (**Supplementary Table 2**). After quality control, the valid sequences were processed to generate representative feature sequences and corresponding feature abundance tables. The number of sequences at each stage for each sample was then summarized (**Supplementary Table 3**). After quality control, feature counts were summarized for each sample to show the sequencing depth and its distribution across the dataset.

#### 3.1.2 Evaluation of species richness and sequencing saturation

To assess species richness across the 30 samples and to confirm that the sequencing depth was sufficient, rarefaction curves were generated. The curves for all groups approached a clear plateau, suggesting that most species present had already been detected and that further sequencing would be unlikely to uncover many additional taxa (**Figure 2A**). Rank abundance curves suggested that the combination group showed noticeably longer and flatter curves, suggesting greater species richness and a more even microbial community structure compared to the control, exercise only, and fucoidan only groups (**Figure 2B**). Petal diagram revealed that 256 features were shared across all groups, representing the core microbial community. Each group also had its own distinct set of unique features, with 22, 13, 44, 6, 17, 17, 9, 12, 11, and 16 found in BFEX0, control, F1, F2, X1, X1F1, X1F2, X2, X2F1, and X2F2, respectively (**Figure 2C**). This pattern of shared core features alongside group-specific variations was further supported by complementary intersection analyses, which confirmed the distinct distribution of microbial features across experimental groups (**Supplementary Figures 1A–C**).

**Figure 2:**
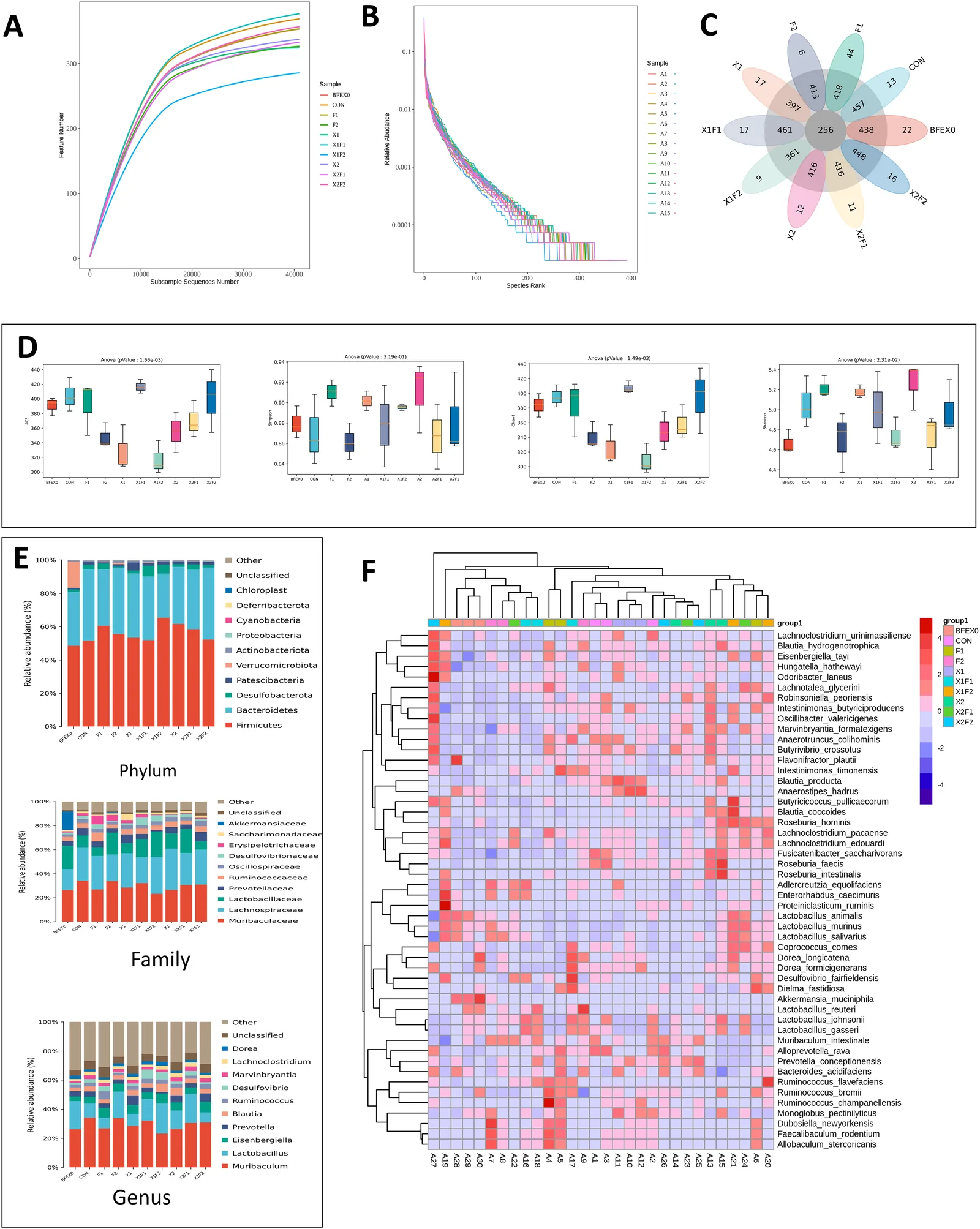
Overview of gut microbiome composition and diversity across experimental groups. **(A)** Rarefaction curves demonstrating sequencing depth and saturation across samples. **(B)** Rank-abundance curves illustrating species richness and community evenness among experimental groups. **(C)** Petal diagram showing shared and unique microbial features, highlighting both the core microbiota and group-specific variations. **(D)** Alpha diversity analysis presented as box plots, including richness indices (ACE and Chao1) and diversity indices (Shannon and Simpson), with significant differences observed across groups (p < 0.001). **(E)** Relative abundance of microbial taxa across experimental groups. **(F)** Heatmap depicting differential abundance and clustering patterns of bacterial taxa in response to exercise (X), fucoidan (F), and their combined interventions.

#### 3.1.3 Evaluation of microbial community variation among groups

To assess how experimental treatments affect gut microbiota composition, we calculated alpha diversity using Chao1 and ACE indices to estimate species richness, and Shannon and Simpson metrics to evaluate overall diversity and evenness. When compared to the control group, the number of detected species was significantly lower under a number of treatment groups, most notably in X1 and X1F2, according to the ACE and Chao1 indices (**Figure 2D**). On the other hand, the X1F1 group showed the highest species richness, even outperforming the control. As a result, groups X1, X1F2, X2, and F2 had much lower richness (Chao1 index) than the control.

Species richness and community balance were found to be strongly decoupled by the Shannon and Simpson indexes. Interestingly, X2, X1, and F1 by themselves produced the highest Shannon and Simpson values, indicating communities with better evenness and variety even though their richness was lower. This suggests that these treatments act as selective pressures, reducing overall species richness while promoting a more balanced and evenly distributed microbial community. F2 was found to be reduced evenness and richness. The BFEX0 treatment showed the lowest Shannon diversity and a lower Simpson index, although it preserved a moderate level of species richness. On the other hand, the combination treatments displayed synergistic or intermediate effects. For example, the greatest reduction in richness, was caused by X1F2, suggesting a synergistic negative effect.

#### 3.1.4 Taxonomy of gut microbiota

Heatmap analysis showed treatment-associated differences in microbial community composition and the relative abundance of specific taxa. Differential abundance analysis was used to identify bacterial taxa that varied across the treatment groups based on their abundance and relative abundance (**Supplementary Datasets 1** and **2**). When comparing the mono-treatment groups (F1, F2, X1, X2) with the combination groups (X1F1, X1F2, X2F1, X2F2), clear differences in the Bacteroidetes-to-Firmicutes (B/F) ratio can be observed. The monotherapy groups showed relatively similar B/F ratios, whereas greater variation was observed among the combination groups. These compositional differences across taxonomic levels were consistently reflected in the broader distribution of microbial taxa from phylum to species level (**Supplementary Figures 3A–F**). In contrast, the combination groups showed greater variation in microbial composition across the taxonomic levels examined. These differences were also reflected in the relative distribution of community features across groups, indicating variation in the contribution of taxa associated with the control and intervention groups (**Supplementary Figure 2**). Genus-level analysis showed treatment-associated differences in microbial composition, including higher relative abundance of *Akkermansia* in the X2F1 and X2F2 groups compared with the control group (**Figure 2E**). In contrast, *Allobaculum* showed lower relative abundance in X2 compared with the control, whereas its abundance was higher in X2F2, indicating a different abundance pattern between these treatment groups. These compositional shifts were further reflected in clustering patterns of differentially abundant taxa, highlighting treatment-specific microbial restructuring across groups (**Figure 2F**). Similar clustering patterns were observed across multiple taxonomic levels, further highlighting treatment-specific shifts in microbial composition (**Supplementary Figures 4A–D**). We observed enrichment of several commensal bacteria and taxa associated with SCFA production across multiple intervention groups. This included higher relative abundance of the butyrate-producing genus *Roseburia*, including *R. hominis* and *R. faecis*, in X2 and X2F1 compared with the control group (**Figure 3F**). The relative abundance of several *Lactobacillus* species, including *L. reuteri*, *L. johnsonii*, *L. gasseri*, *L. murinus*, and *L. animalis*, varied across the treatment groups. In X2F1, *L. johnsonii* and *L. gasseri* were significantly decreased compared with the control (p < 0.05), whereas *L. murinus* was significantly increased (p < 0.01). The mucin-degrading bacterium *Akkermansia muciniphila* was also significantly increased in BFEXO compared with the control (p < 0.0001).

**Figure 3:**
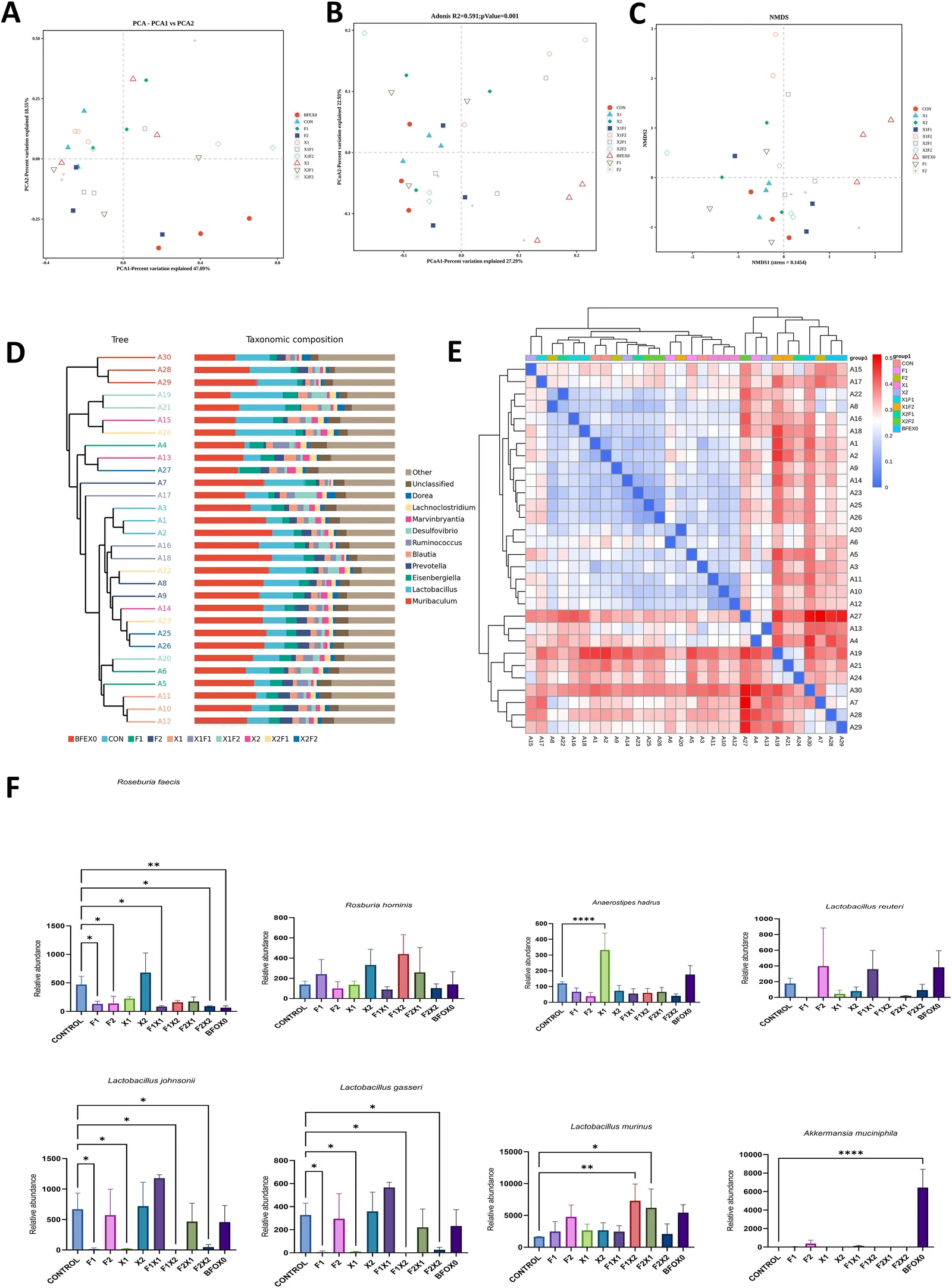
Beta diversity and differential abundance analysis of gut microbial communities across experimental groups. **(A)** Principal Component Analysis (PCA) illustrating variation in microbial community composition, where closer samples indicate greater similarity. **(B)** Principal Coordinate Analysis (PCoA) based on Bray–Curtis dissimilarity, demonstrating clear separation between groups, with PERMANOVA confirming statistical significance (R² = 0.291, p = 0.001). **(C)** Non-metric Multidimensional Scaling (NMDS) showing community dissimilarity, with a stress value < 0.2 indicating a reliable representation of the data. **(D)** Hierarchical clustering dendrogram based on Bray–Curtis distances, revealing distinct grouping patterns among samples. **(E)** Heatmap of Bray–Curtis dissimilarity, where color intensity reflects the degree of difference between microbial communities. **(F)** Differential abundance analysis of selected beneficial and short-chain fatty acid–producing taxa across treatment groups relative to control, presented as log-fold changes (mean ± SD). Statistical comparisons were made between each treatment group and the control using one-way ANOVA followed by Dunnett’s multiple-comparisons test. Only statistically significant comparisons are shown (*p < 0.05, **p < 0.01, ***p < 0.001. ****p < 0.0001).

#### 3.1.5 Distribution of beta diversity

To assess differences in microbial community structure among the control, X1, X2, F1, F2, and combination groups, beta diversity analysis was performed using Bray–Curtis dissimilarity and visualized through PCA, PCoA, and NMDS ordinations (**Figures 3A–C**). PCA showed that the first principal component (PC1) explained 47.09% of the total variance, while the second component (PC2) accounted for 18.55%, indicating that samples in closer proximity shared more similar microbial compositions. PCoA showed separation in microbial community composition among the experimental groups, with PERMANOVA confirming a significant overall group effect (R² = 0.291, p = 0.001). Consistently, NMDS analysis yielded a stress value below 0.2, indicating a reliable representation of the data. Hierarchical clustering based on Bray–Curtis dissimilarity separated the microbial communities into two major clusters (**Figure 3D**). One cluster was characterized by a more diverse microbial composition enriched in *Muribaculum*, whereas the other was predominantly dominated by *Lactobacillus*. Notably, the *Lactobacillus*-dominant cluster was primarily associated with the X1F2 and X2F1 combination treatment groups (**Figure 3E**). The Bray–Curtis distance heatmap showed patterns of within- and between-group similarity in microbial community composition. ANOSIM revealed a significant overall difference in microbial community composition among the experimental groups (Global R = 0.48, p < 0.001), indicating that between-group dissimilarities were greater than within-group dissimilarities (**Figure 4A**). Furthermore, PLS-DA provided an additional visualization of group-associated patterns in microbial community composition (**Figure 4B**).

**Figure 4:**
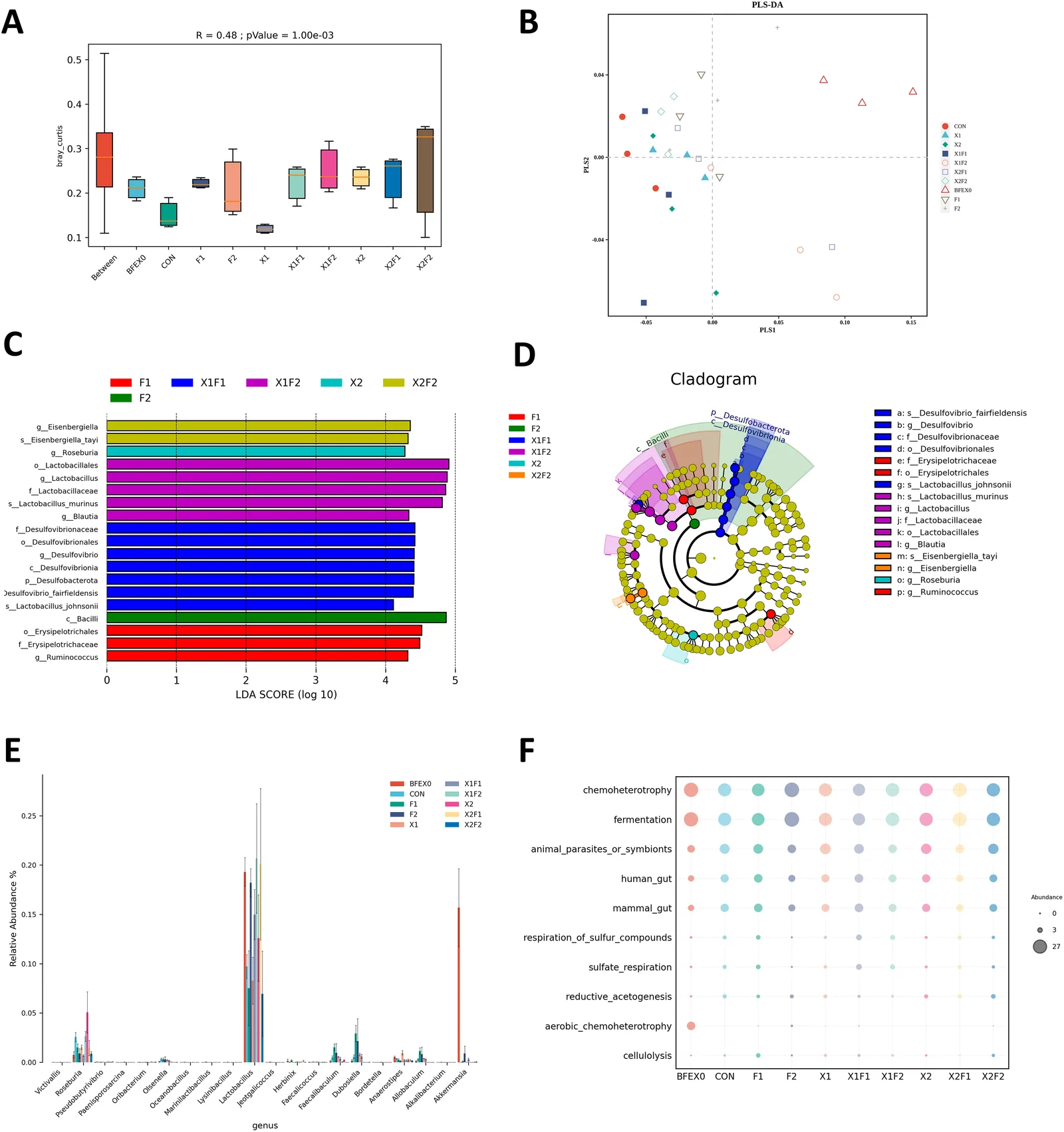
Multivariate and biomarker analysis of gut microbial communities across experimental groups. **(A)** ANOSIM analysis demonstrating significant differences in microbial community composition between groups (Global R = 0.48, p < 0.001). **(B)** Partial Least Squares Discriminant Analysis (PLS-DA) showing clear separation among experimental groups, with tight clustering within groups indicating high similarity and distinct microbial profiles. **(C)** Histogram of Linear Discriminant Analysis (LDA) scores highlighting taxa with significant effect sizes (LDA score > 4.0). **(D)** LEfSe cladogram illustrating phylogenetic relationships of differentially abundant taxa, with circle size corresponding to relative abundance. **(E)** Genus-level abundance profiles of selected bacterial taxa across experimental groups. **(F)** Heatmap of predicted microbial functional pathways based on FAPROTAX, indicating enrichment of sulfate respiration pathways in X1-related groups.

LEfSe analysis and cladogram was employed to identify specific bacterial taxa that were differentially abundant between experimental groups and to quantify the effect size of these differences (**Figures 4C, D**). The analysis revealed distinct, group-specific microbial biomarkers. LEfSe identified *Eisenbergiella*, particularly *E. tayi*, as a discriminating taxon for X2F2 (LDA score > 4.5). In contrast, X1F2 was characterized by *Lactobacillales*, *Lactobacillus*, *L. murinus*, and *L. johnsonii* as discriminating taxa (LDA scores > 4.0). Furthermore, the X1F1 group was defined by a notable enrichment of sulfate-reducing bacteria, including the phylum *Desulfobacterota* and the genus *Desulfovibrio*. The group F2 was uniquely associated with the genus *Blautia*, while members of the order *Erysipelotrichales* and the genus *Ruminococcus* served as biomarkers for other groups.

A Kruskal-Wallis rank-sum test was performed on genus-level taxonomic abundances to identify taxa that were differentially abundant across the experimental groups (**Figure 4E**). Kruskal–Wallis analysis identified significant overall differences in the abundance of selected genera, including *Akkermansia, Lactobacillus*, and *Anaerostipes* (**Figure 4E**). The functional potential of the microbial communities, predicted using FAPROTAX, revealed distinct treatment-driven shifts in metabolic pathways (**Figure 4F**). Consistent with the taxonomic patterns, X1-related groups showed a higher predicted abundance of sulfate respiration pathways, indicating a greater microbial potential for hydrogen sulfide production. In contrast, Fermentation-associated functions were also more highly represented in several intervention groups, suggesting differences in predicted fermentative potential. The core functional profile of a mammalian gut community (chemoheterotrophy, human gut) was maintained across all samples, confirming the gut-like nature of the communities while highlighting the specific metabolic alterations induced by the treatments.

### 3.2 Metabolomics analysis

#### 3.2.1 Intersection analysis reveals key modulatory effects

To quantify the similarity between the control and each experimental group, we analyzed the size of their intersections, which represents the number of shared elements between them. The results, ranked from largest to smallest intersection size, are presented in **Figure 5A**. X2 showed the smallest number of metabolite features shared with the control in the intersection analysis (**Figure 5A**).

**Figure 5:**
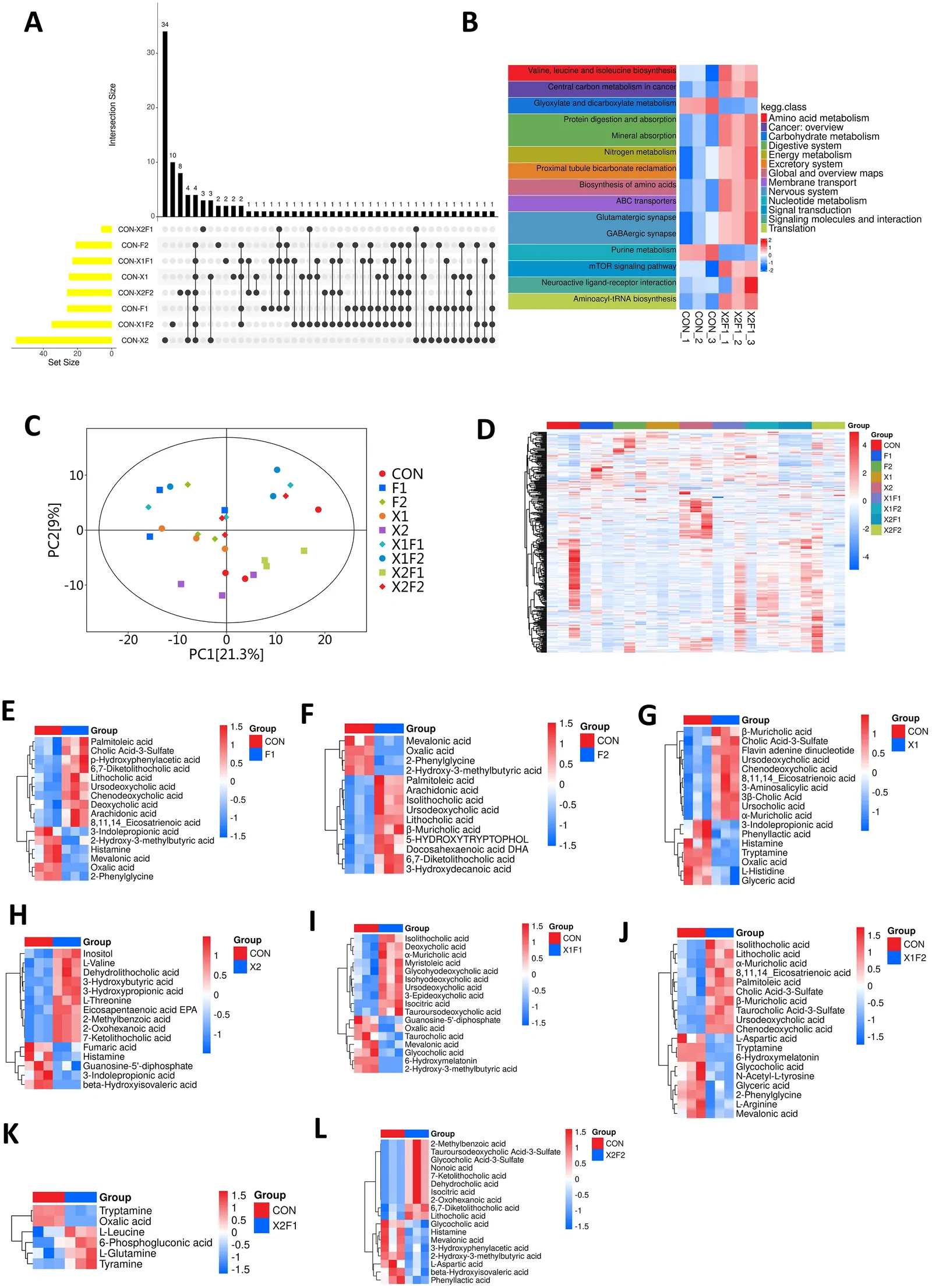
Integrated metabolomic profiling and comparative analysis across experimental groups. **(A)** UpSet plot illustrating shared and unique metabolite features across experimental groups, with bar plots indicating the size of intersections relative to the control. **(B)** Functional profile of metabolites shared between the control and X2F1 groups. **(C)** Principal Component Analysis (PCA) score plot of the fecal metabolome, showing global metabolic variation among groups; PC1 and PC2 explain 21.3% and 9.0% of the total variance, respectively. **(D)** Heatmap depicting key metabolites modulated by exercise and fucoidan interventions. **(E–L)** Comparative metabolomic heatmaps representing group-specific metabolic alterations in F1 **(E)**, F2 **(F)**, X1 **(G)**, X2 **(H)**, X1F1 **(I)**, X1F2 **(J)**, X2F1 **(K)**, and X2F2 **(L)**, highlighting metabolites significantly changed (p < 0.05) relative to the control group.

KEGG pathway enrichment analysis identified treatment-associated differences in metabolic and signaling pathways. In X2F1, differential metabolites were enriched in pathways related to amino acid and energy metabolism. This was accompanied by coordinated changes in digestive and signaling pathways, including mTOR and neurotransmission-related processes (**Figure 5B**). These pathway-level changes suggest a systemic shift in metabolic function under combined treatment. Integrated pathway-level analyses further supported these findings, revealing coordinated relationships among differential metabolites and enriched biological pathways (**Supplementary Figures 10A–E**). Functional distribution of metabolites across pathways also highlighted treatment-specific metabolic organization (**Supplementary Figures 11A–G**). Unsupervised principal component analysis (PCA) showed that the first two principal components, PC1 and PC2, explained 21.3% and 9.0% of the total variance, respectively (**Figure 5C**). The heatmap showed distinct patterns of fecal metabolite abundance across the experimental groups (**Figure 5D**) suggests that the combined exercise and fucoidan intervention modulates the metabolic profile. Comparative metabolomic profiling demonstrated group-specific metabolic signatures (**Supplementary Dataset 3**), with distinct patterns of metabolite regulation observed across all intervention groups (**Figures 5E–L**). In F1, several bile acid-related metabolites, including lithocholic acid, cholic acid-3-sulfate, deoxycholic acid, chenodeoxycholic acid, and ursodeoxycholic acid, were increased, whereas 3-indolepropionic acid, histamine, mevalonic acid, oxalic acid, and 2-hydroxy-3-methylbutyric acid were decreased. F2 showed increases in metabolites including docosahexaenoic acid, 5-hydroxytryptophol, and 3-hydroxydecanoic acid, together with decreases in mevalonic acid, oxalic acid, and 2-hydroxy-3-methylbutyric acid. The exercise groups also showed significant metabolite changes relative to control. X1 was associated with increased abundance of several bile acid-related metabolites and flavin adenine dinucleotide, while histamine, 3-indolepropionic acid, L-histidine, glyceric acid, oxalic acid, and tryptamine were decreased. In X2, L-valine, L-threonine, eicosapentaenoic acid, inositol, and 3-hydroxybutyric acid were increased, whereas fumaric acid, histamine, 3-indolepropionic acid, guanosine-5′-diphosphate, and beta-hydroxyisovaleric acid were decreased. Among the combination groups, X1F1 showed increases in several bile acid derivatives, including isohyodeoxycholic acid, glycohyodeoxycholic acid, tauroursodeoxycholic acid, and ursodeoxycholic acid, while taurocholic acid, glycocholic acid, mevalonic acid, oxalic acid, and 6-hydroxymelatonin were decreased. X1F2 showed increased abundance of several bile acid-related metabolites, whereas N-acetyl-L-tyrosine, L-aspartic acid, L-arginine, mevalonic acid, glyceric acid, tryptamine, and 6-hydroxymelatonin were decreased. In X2F1, oxalic acid and tryptamine were decreased, while tyramine, 6-phosphogluconic acid, L-leucine, and L-glutamine were increased relative to control. X2F2 showed increases in several bile acid-related metabolites, nonanoic acid, and isocitric acid, together with decreases in L-aspartic acid, histamine, mevalonic acid, glycocholic acid, phenyllactic acid, and beta-hydroxyisovaleric acid. Across the intervention-versus-control comparisons, mevalonic acid and histamine were repeatedly identified among the altered metabolites, while bile acid-related metabolites represented a prominent component of several treatment-associated profiles. These patterns were further supported by the distribution, clustering, fold-change, and radar analyses (**Supplementary Figures 6**-**9**). Correlation analysis further identified associations between dominant bacterial genera and selected metabolite pathways across experimental groups (**Supplementary Figures 5A–I**).

### 3.3 Effect of exercise and fucoidan on tumor burden

#### 3.3.1 Tumour growth, volume and its weight

Subcutaneously injected MC38 tumor growth in mice was evaluated by measuring final tumor volume and weight after 28 days. Compared to the control group, monotherapy treatments F1 (p < 0.001) and X2 (p < 0.001) significantly reduced tumor weight (**Figure 6A**). Similarly, among the combination treatments, both X1F2 (p < 0.01) and X2F1 (p < 0.001) showed a significant reduction in tumor weight compared to the control. After 28 days, tumor volume was also significantly decreased in the X2F1 group (p < 0.0001; **Figure 6B**) compared to control. Although a similar trend of reduced tumor growth was observed in other combination groups (**Figure 6C**), these differences did not reach statistical significance. Body weight, food intake, and water consumption were monitored across all groups. No significant differences were observed among groups for any of these parameters (**Supplementary Figures 13A–C**), suggesting that the observed treatment effects were not associated with differences in body weight, food intake, or water consumption.

**Figure 6:**
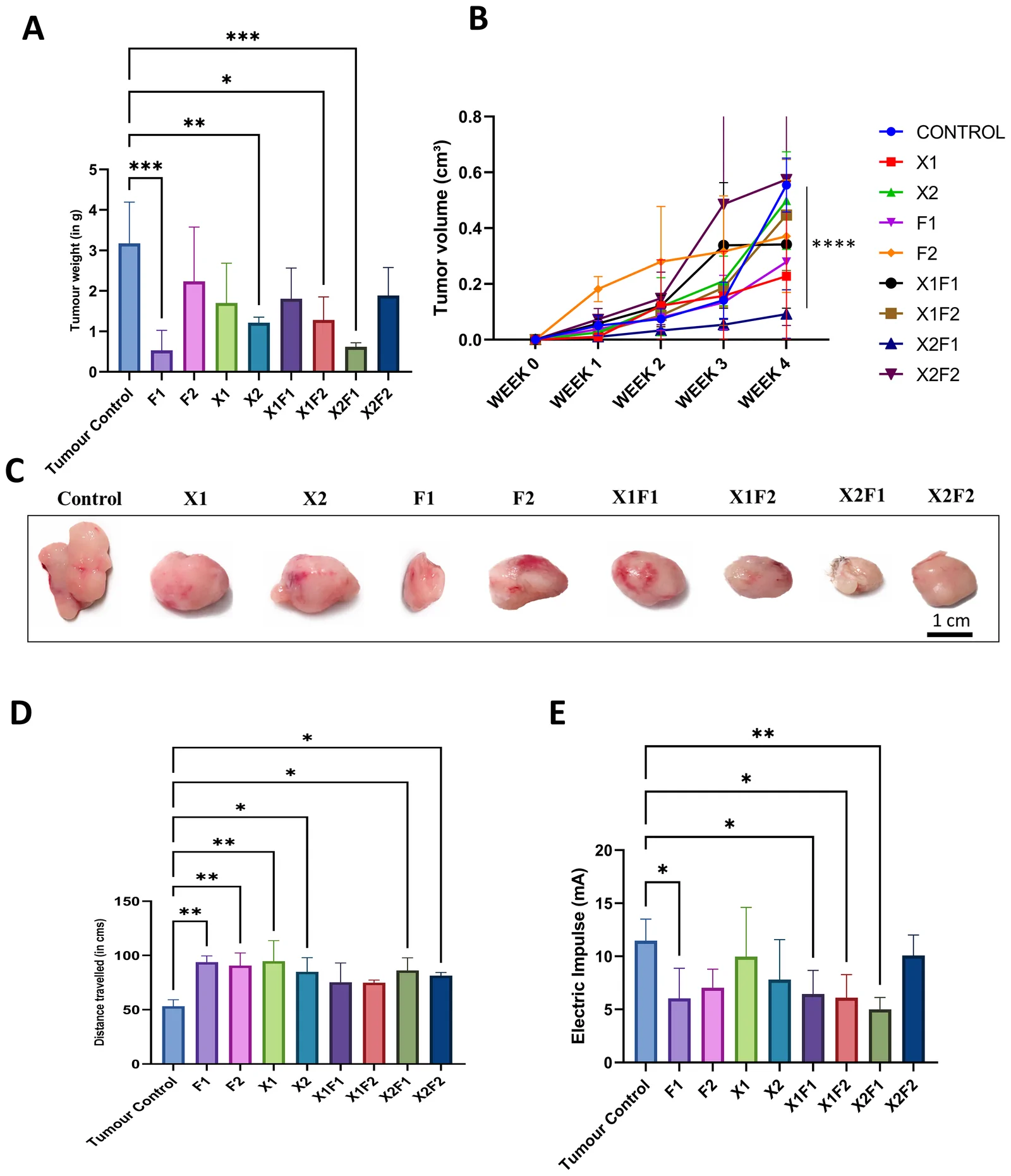
Effects of exercise and fucoidan on tumor progression and endurance. **(A)** Tumor weight measured at the experimental endpoint. **(B)** Tumor volume monitored over four weeks after MC38 inoculation. **(C)** Representative images of excised tumors from each experimental group. **(D)** Endurance performance assessed by distance traveled during the run-to-fatigue test. **(D)** Electric stimulus requirement during treadmill testing. Data are presented as mean ± SD. Statistical comparisons were made between each intervention group and the tumor-bearing control. Only statistically significant comparisons are shown (*p < 0.05, **p < 0.01, ***p < 0.001, **p < 0.0001).

#### 3.3.2 Endurance test

Following 28 days of cancer induction, locomotor performance was evaluated using a forced treadmill test, where mild electric stimuli were applied to sustain running. In the distance travelled, tumour control animals exhibited the lowest performance, indicating a marked decline in endurance and physical capacity during cancer progression. In contrast, monotherapy with F1 (p<0.01), F2 (p<0.01), X1 (p<0.01), and X2 (p<0.05) resulted in a significant increase in running distance (**Figure 6D**). Similarly, X2F1 and X2F2 also demonstrated significant improvements compared to tumor control (p< 0.05). In the electric impulse parameter, tumor control animals required the highest level of stimulation, indicating reduced intrinsic motivation to run and greater fatigue-related disengagement. In contrast, several treatment groups, including F1 (p<0.05), X1F1(p<0.05), X1F2 (p<0.05), and X2F1(p<0.01) showed significantly lower impulse requirements (**Figure 6E**).

#### 3.3.3 Gene expression analysis

qRT-PCR analysis revealed significant modulation of apoptosis and proliferation-related genes across treatment groups (**Figure 7**). Expression of p53 was significantly increased in F1 (p<0.0001), F2 (p<0.05), X1F1 (p<0.001), X1F2 (p<0.0001), X2F1 (p<0.0001), and X2F2 (p<0.0001). Consistently, Bax expression was significantly (p<0.0001) elevated in F1, X1F1, X1F2, X2F1, and X2F2. In contrast, the anti-apoptotic gene Bcl-2 was significantly reduced in X1 (p<0.05) and more markedly in X2F1 (p<0.001). Regarding proliferation, Ki67 expression was significantly decreased in X1 (p<0.01), X2 (p<0.001), F1 (p<0.0001), F2 (p<0.01), X1F2 (p<0.01), X2F1 (p<0.0001), and X2F2 (p<0.05). VEGF expression showed limited changes, with significant reduction observed only in X2 (p<0.05) and F1 (p<0.001), while other groups remained non-significant.

**Figure 7:**
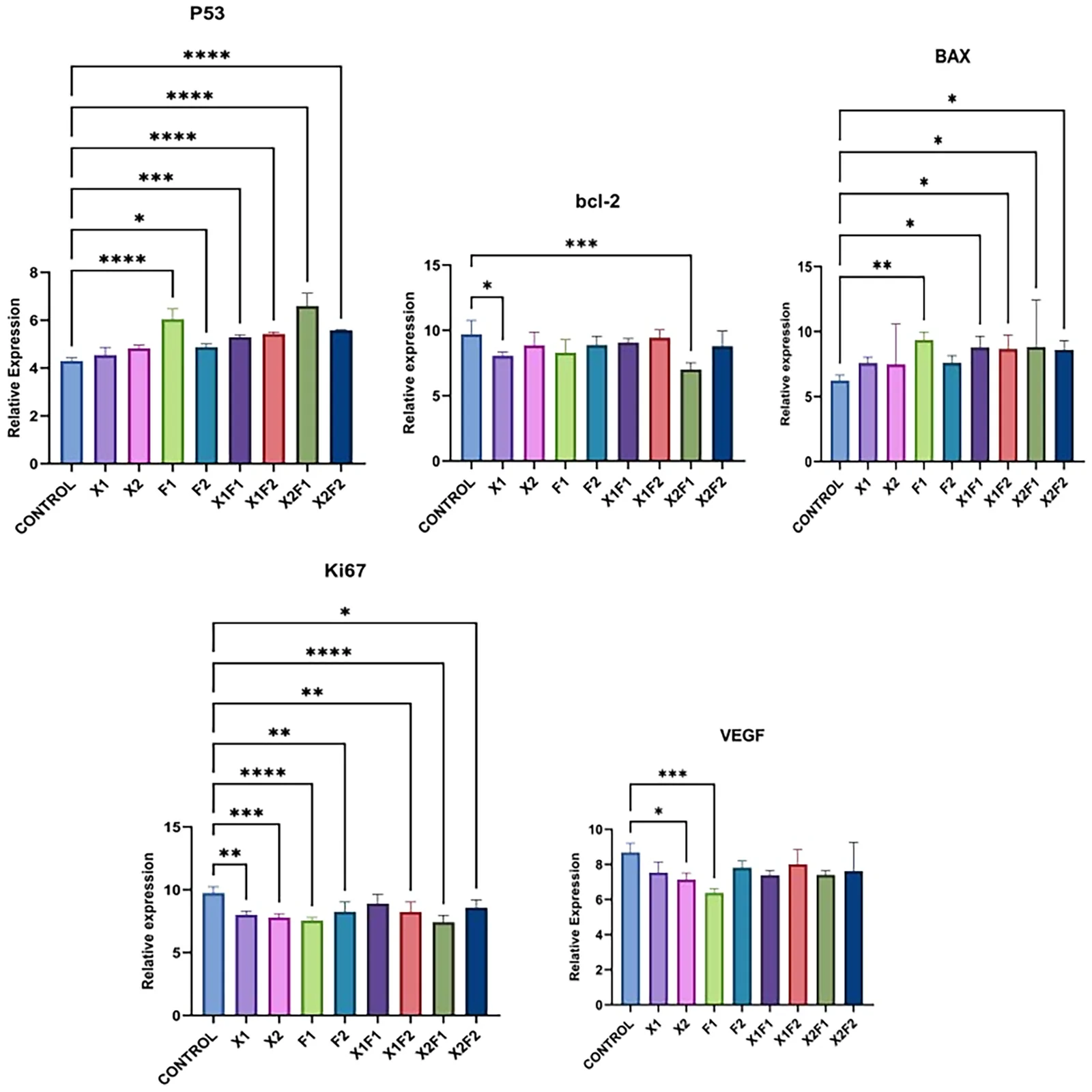
Relative gene expression analysis in tumor tissues across experimental groups. Relative mRNA expression levels of apoptosis- and proliferation-related genes (*p53, Bax, Bcl-2, Ki67*, and *VEGF*) in tumor tissues from control and treated groups (exercise and fucoidan). Data are presented as mean ± SD from triplicate experiments. Statistical comparisons were made between each intervention group and the control. Only statistically significant comparisons are shown (*p < 0.05, **p < 0.01, ***p < 0.001. ****p < 0.0001).

#### 3.3.4 Histological analysis

Histological examination of intestinal tissues revealed minimal structural alterations in the tumor control group following subcutaneous MC38 induction. Mild disruptions in mucosal architecture were observed in control group, indicating a limited impact of tumor burden on intestinal integrity. In contrast, all treatment groups, including F1, F2, X1, X2, and the combination groups, maintained normal intestinal morphology with well-preserved epithelial structure and intact villi (**Supplementary Table 5**).

## 4 Discussion

The present study indicates that dietary fucoidan, alone and in combination with exercise, was associated with changes in gut microbial composition, fecal metabolite profiles, tumor-related outcomes, and gene expression in the MC38 model. Rather than being explained simply by changes in overall microbial diversity, the responses appeared to involve selective changes in microbial taxa and their associated metabolic profiles. Beyond its microbiome-mediated actions, fucoidan has also been widely reported to exert direct anticancer effects across multiple cancer types, including colorectal cancer, through activation of apoptotic pathways, caspase signaling, suppression of antiapoptotic proteins, and induction of oxidative stress–mediated cell death. These complementary mechanisms suggest that fucoidan acts through both microbial and host-mediated pathways (Mensah et al., 2023). Consistent with this, among all interventions, the combination of moderate exercise with low-dose fucoidan (X2F1) produced the most consistent improvements across microbial, metabolic, and tumor-related outcomes, indicating that the observed benefits likely arise from coordinated interactions rather than isolated effects.

A key finding is that tumor suppression was not linked to increased microbial diversity alone, but rather to selective restructuring of the microbial community. While alpha diversity indices varied across groups, they did not consistently align with tumor outcomes. Instead, the X2F1 group exhibited a more functionally organized microbial profile, particularly the enrichment of *L. murinus* alongside modulation of taxa associated with SCFA production. *L. murinus* has been shown to reduce inflammation by suppressing IL-8 production, improve gut barrier integrity, and lower endotoxin levels, thereby mitigating microbiota-induced intestinal inflammation (Pan et al., 2018). Several *Lactobacillus* species are known to promote SCFA production, reduce harmful metabolites, and enhance host immune activity (Markowiak-Kopeć and Śliżewska, 2020). This supports the view that microbial function and metabolic output are more critical determinants of host outcomes than diversity alone (Louis et al., 2014). The selective enrichment of specific taxa further emphasizes the importance of strain-level specificity in shaping biological responses.

Although X2F1 also showed significant differences from the control across several microbial, metabolic, tumor-related, and gene-expression outcomes, these findings do not demonstrate that exercise potentiated the effect of fucoidan or that the combination acted synergistically. We therefore interpret X2F1 as a combination associated with a broad treatment response rather than as evidence of a superior or synergistic effect. Exercise-induced changes in the gut microbiome can vary substantially with exercise intensity, duration, host physiology, and dietary background, which may partly explain the heterogeneous responses observed across treatment groups (Boytar et al., 2022). It could be speculated that the comparatively broad response observed in X2F1 relative to the control may reflect the interaction of low-dose fucoidan with the physiological changes associated with X2 exercise. Fucoidan bioactivity is influenced by factors such as molecular weight, monosaccharide composition, sulfation pattern, source, and extraction method, while exercise responses are similarly dependent on intensity and host metabolic status. Thus, different combinations of dietary substrate availability and exercise-induced physiological changes may generate distinct microbial and metabolic environments. However, confirmation of a true interaction between fucoidan and exercise will require a factorial analysis specifically testing the interaction between fucoidan and exercise. This interpretation is supported by evidence indicating that dietary substrates exert direct and dominant effects on gut microbial composition and metabolism, whereas exercise-induced changes are generally more variable and dependent on host physiological context (Flint et al., 2012; Kang et al., 2014; Yun et al., 2022).

The metabolomic findings add an important functional dimension to the microbial changes observed in this study. In X2F1, significant differences relative to the control involved metabolites associated with amino acid and central carbon metabolism, including changes in tryptamine, L-glutamine, L-leucine, and 6-phosphogluconic acid. These findings should not be interpreted as direct evidence of pathway activation, but rather as a broader shift in fecal metabolic organization. This is biologically relevant because colorectal cancer is accompanied by extensive metabolic rewiring involving glycolysis, glutamine utilization, lipid metabolism, redox balance, and signaling pathways such as PI3K/AKT/mTOR. Fucoidan has also been reported to influence cellular bioenergetics, carbohydrate and protein metabolism, mitochondrial function, and apoptosis-related signaling (Jin et al., 2022; Shiau et al., 2022). The changes in L-glutamine, L-leucine, and 6-phosphogluconic acid are therefore better interpreted as indicators of altered nutrient and energy metabolism rather than as individually beneficial metabolites. Likewise, changes in tryptophan-related metabolites, bile acids, and other microbial products may contribute to the broader metabolic environment associated with colorectal tumor development. Notably, X2F1 was characterized by changes in amino acid and energy-related metabolism, whereas other treatment groups showed different metabolite patterns, including prominent bile acid-related changes.

The tumor gene-expression findings provide an additional host-level correlate of these responses. Increased p53 and Bax expression together with reduced Bcl-2 and Ki67 in selected intervention groups is consistent with apoptosis-related and anti-proliferative regulation. However, because these measurements were based on mRNA expression, they should not be interpreted as direct demonstration of apoptosis or pathway activation. Thus microbial and metabolomic findings support a model in which dietary and exercise interventions are associated with coordinated changes across the gut microbiome, metabolome, and tumor-related molecular responses. In addition to tumor-related outcomes, the preservation of physical performance suggests improved systemic resilience under tumor burden.

An additional consideration is that the present study used isolated fucoidan rather than whole brown seaweed, which is important when considering nutritional translation. Fucoidan is a sulfated, poorly digestible polysaccharide that can reach the colon and be utilized by gut microorganisms, thereby influencing microbial composition and metabolite production (Flint et al., 2012; Yuan et al., 2021). Previous studies have likewise shown that fucoidan supplementation can alter beneficial microbial taxa and microbiota-associated metabolic pathway (Li et al., 2024). However, whole brown seaweed provides a much more complex food matrix containing alginate, laminarin, phlorotannins, fucoxanthin, proteins, fatty acids, vitamins, and minerals (Khammassi et al., 2026). Because different polysaccharide fractions and phytochemicals may be selectively utilized or transformed by the gut microbiota, whole-seaweed consumption could produce additive, synergistic, or even antagonistic effects that differ from those of purified fucoidan alone (Jiang et al., 2024). Conversely, the use of isolated fucoidan in the present study allows its contribution to be examined with greater specificity. Future studies comparing purified fucoidan with whole-seaweed preparations will be important for determining how the food matrix modifies microbiome, metabolic, and host responses. Future translation should therefore focus on standardized fucoidan preparations with clearly defined molecular weight, sulfate content, source, purity, and dose. A factorial clinical or preclinical design incorporating fucoidan alone, exercise alone, and their combination would be particularly valuable because it would allow formal testing of the interaction between the two interventions rather than inferring enhancement from treatment-versus-control comparisons. Serial microbiome and metabolome measurements before and after tumor development would also help determine whether the pre-induction microbial changes observed here persist during tumor progression. In humans, these studies could incorporate fecal metabolomics, microbial profiling, circulating inflammatory and metabolic markers, and clinically relevant outcomes while accounting for habitual diet and physical activity. Exercise is already associated with reduced CRC risk and may influence the gut microbiome, but microbiome-mediated mechanisms in patients with cancer remain insufficiently established.

The microbiome and metabolome analyses in the present study were restricted to the pre-induction period. This design allowed us to characterize the microbial and metabolic environment established by six weeks of intervention before tumor challenge, but it does not determine whether these changes persisted after tumor development or directly mediated the subsequent tumor response. Future studies incorporating longitudinal post-induction multi-omics analysis, mechanistic microbiota manipulation, and formal interaction testing will therefore be necessary to determine whether particular fucoidan–exercise combinations produce genuinely additive or synergistic effects.

## 5 Conclusion

This study highlights brown seaweed-derived fucoidan as a marine sulfated polysaccharide associated with coordinated changes in the gut microbiome, fecal metabolome, and tumor-related responses in a murine colorectal cancer model. Fucoidan-containing interventions altered microbial composition, including enrichment of *Lactobacillus murinus*, together with changes in amino acid, bile acid, and central carbon metabolism. These microbial and metabolic alterations occurred alongside reduced tumor burden and changes in apoptosis- and proliferation-related gene expression, including modulation of p53, Bax, Bcl-2, and Ki67. Low-dose fucoidan showed a clear treatment-associated response across several measured outcomes, while the X2F1 combination was also characterized by broad microbial, metabolic, and tumor-related changes relative to the control. Thus, the findings suggest that dietary fucoidan, alone or in combination with exercise, is associated with coordinated remodeling of the gut microbiome, metabolic profile, and tumor-related molecular responses during colorectal cancer development. This work supports further investigation of fucoidan as a microbiome-associated marine bioactive compound with potential relevance to nutritional strategies for colorectal cancer.

## Data Availability

The data presented in the study are deposited in the NCBI Bioproject repository, accession number PRJNA1470399.
