# Supplementary material for: Effects of dietary fucoidan and exercise on the gut microbiome, fecal metabolome, and tumor-related responses in a murine colorectal cancer model
Source: Front Cell Infect Microbiol. 2026 Sep 18;16:1926528. doi: 10.3389/fcimb.2026.1926528 (PMC13630683; doi:10.3389/fcimb.2026.1926528)
Supplement: Supplementary file 1 [file SupplementaryFile1.docx]

**Supplementary Fig. 1 Analysis of shared and unique microbial features across groups.**
(A) Venn diagram illustrating the distribution of shared and unique features among different groups, where overlapping regions represent common features and non-overlapping regions indicate group-specific features. (B) Bar plot showing the total number of features identified in each group. (C) UpSet plot depicting the intersection and distribution of feature counts across the respective experimental groups.

**Supplementary Fig. 2**: **Ternary phase diagram showing the relative distribution of microbial attributes.** The positioning of samples within the diagram reflects the dominance or balance of attributes associated with BFEX0 and CON in comparison to the intervention groups.

**Supplementary Fig. 3 Taxonomic distribution of microbial communities across different levels.** Histograms representing the relative abundance of microbial taxa across experimental groups at multiple taxonomic levels: (A) phylum, (B) class, (C) order, (D) family, (E) genus, and (F) species. These plots illustrate variations in microbial composition and distribution across groups at increasing levels of taxonomic resolution.

**Supplementary Fig. 4 Heatmap showing clustering of microbial abundance across taxonomic levels** the relative abundance and clustering patterns of microbial taxa across experimental groups at different taxonomic levels: (A) phylum, (B) order, (C) family, and (D) genus. The clustering highlights similarities and differences in microbial composition among groups based on taxonomic profiles.

**Supplementary Fig. 5. Spearman correlation analysis between microbial genera and metabolite pathways.** Heatmaps showing Spearman rank correlations between selected microbial genera and metabolite superpathways across experimental groups: (A) Control, (B) X1, (C) X2, (D) F1, (E) F2, (F) X1F1, (G) X1F2, (H) X2F1, and (I) X2F2. Metabolites were annotated using KEGG identifiers and grouped into superpathways, with correlations summarized at the pathway level. The analysis includes the top 20 genera and top 20 metabolite pathways based on variability and abundance.

**Supplementary Fig. 6. Distribution of metabolite levels across experimental groups.**
Box and whisker plots illustrating the variation in metabolite levels across different groups: (A) X1, (B) X2, (C) F1, (D) F2, (E) X1F1, (F) X1F2, (G) X2F1, and (H) X2F2, compared with the control (CON). Statistical significance for each metabolite was determined relative to the control group using Student’s t-test. Differences between groups are indicated by distinct colors. Selected metabolites, particularly in the F2 group, showed significant upregulation compared to control. A p-value of <0.05 was considered statistically significant.

**Supplementary Fig. 7 K-means clustering analysis of metabolite profiles across experimental groups.** K-means clustering heatmap showing patterns of metabolite abundance across sample groups, where the horizontal axis represents experimental groups and the vertical axis represents normalized metabolite levels. Data were standardized using Z-score transformation prior to clustering. Each cluster represents a group of metabolites exhibiting similar trends across treatments, and the number of metabolites within each cluster is indicated.

**Supplementary Fig. 8 Matchstick analysis of differential metabolite expression across experimental groups.** Matchstick plots representing relative changes in metabolite levels across groups: (A) F1, (B) F2, (C) X1, (D) X2, (E) X1F1, (F) X1F2, (G) X2F1, and (H) X2F2, compared with the control (CON). Metabolite ratios were log-transformed (<2) to visualize fold changes, and dot color intensity reflects variable importance in projection (VIP) scores. The analysis highlights metabolites that are significantly upregulated or downregulated in each group. Statistical significance is indicated as *p<0.05, **p<0.01, and ***p<0.001.

**Supplementary Fig. 9 Radar plot illustrating differential metabolite profiles across experimental groups.** Radar charts depicting the relative changes in metabolite levels across groups: (A) F1, (B) F2, (C) X1, (D) X2, (E) X1F1, (F) X1F2, (G) X2F1, and (H) X2F2. Values represent log₂-transformed ratios of metabolite abundance relative to control, as indicated along the axes. Each grid line corresponds to fold-change levels, and the shaded region reflects the overall differential pattern of metabolites within each group. The top 10 metabolites, selected based on statistical significance, are displayed to highlight key metabolic alterations.

**Supplementary Fig. 10 Integrated metabolomic and pathway analysis of combination treatment groups.** (A) Correlation heatmap showing relationships among differential metabolites, where color indicates Pearson correlation (red, positive; blue, negative) and intensity reflects correlation strength; asterisks denote statistical significance. (B) KEGG pathway classification illustrating the distribution of annotated differential metabolites across functional pathways. (C) Bubble plot representing pathway enrichment and topology, where bubble size corresponds to pathway impact and color (−ln p) indicates enrichment significance. (D) KEGG enrichment analysis displaying pathways ranked by Rich factor (x-axis) and pathway name (y-axis), with dot size representing metabolite counts and color indicating statistical significance (Fisher’s exact test). (E) Network diagram depicting interactions among differential metabolites across the combination groups (X1F1, X1F2, X2F1, and X2F2).

**Supplementary Fig. 11.**  **KEGG pathway-based heatmap of shared metabolite profiles across experimental groups.** Heatmaps illustrating the functional distribution of metabolites. (A) X1, (B) X2, (C) F1, (D) F2, (E) X1F1, (F) X1F2, (G) X2F2

**Supplementary Fig. 12 Central carbon metabolism in cancer highlighting metabolite alterations in the X2F1 group.** KEGG pathway map of central carbon metabolism in cancer illustrating the differential regulation of key metabolites identified in the X2F1 group. Metabolites altered under the X2F1 intervention are highlighted to indicate their involvement in major metabolic pathways, including glycolysis, the tricarboxylic acid (TCA) cycle, and amino acid metabolism. Downregulated metabolites such as tryptamine and oxalic acid, along with downregulated metabolites including L-leucine, L-glutamine, and 6-phosphogluconic acid, are mapped onto the pathway to demonstrate coordinated metabolic reprogramming. These alterations suggest a shift away from tumor-supportive metabolic processes, potentially contributing to the reduced tumor burden observed in the X2F1 group.

**Supplementary Fig. 13** **Physiological parameters and intestinal histology following exercise and fucoidan intervention.** (A) Average body weight, (B) water intake, and (C) food consumption measured over the 6-week intervention period. (D) Representative histological images of intestinal tissue stained with hematoxylin and eosin (H&E), showing structural features including goblet cells (GC), lamina propria (LP), intestinal glands (IG), and columnar epithelial cells (CEC).

| Analyze the content | Software/database | version | References or URLs |
| --- | --- | --- | --- |
| Data quality control | Porechop | 0.2.3 | <https://github.com/rrwick/Porechop> |
| Data quality control | Trimmomatic | 0.39 | Bolger AM, Lohse M, Usadel B. Trimmomatic: a flexible trimmer for Illumina sequence data. Bioinformatics. 2014 Aug 1; 30(15):2114-20. <http://www.usadellab.org/cms/index.php?page=trimmomatic> |
| Data quality control | cutadapt | 3.5 | Martin, Marcel. “Cutadapt removes adapter sequences from high-throughput sequencing reads.” EMBnet.journal 17 (2011): 10-12. <https://cutadapt.readthedocs.io/en/stable/index.html> |
| Characterization analysis | qiime2 | 2022.2 | Bolyen E, Rideout JR, Dillon MR, et al. Reproducible, interactive, scalable and extensible microbiome data science using QIIME 2 [published correction appears in Nat Biotechnol. 2019 Sep; 37(9):1091. <https://qiime2.org/> |
| Characterization analysis | lastal | lastal 1268 | Kiełbasa SM, Wan R, Sato K, et al. Adaptive seeds tame genomic sequence comparison. Genome Res. 2011; 21(3):487-493. <https://github.com/mcfrith/last-genome-alignments> |
| Characterization analysis | dada2 | 1.22.0 | Callahan, B., McMurdie, P., Rosen, M. et al. DADA2: High-resolution sample inference from Illumina amplicon data. Nat Methods 13, 581–583 (2016). <https://github.com/benjjneb/dada2> |
| Characterization analysis | vsearch | 2.7.0 | Rognes T, Flouri T, Nichols B, et al. VSEARCH: a versatile open source tool for metagenomics. PeerJ. 2016; 4:e2584. <https://github.com/torognes/vsearch> |
| Species taxonomy | Krona | 2.8.1 | Ondov, B.D., Bergman, N.H. & Phillippy, A.M. Interactive metagenomic visualization in a Web browser. BMC Bioinformatics 12, 385 (2011). <https://github.com/marbl/Krona> |
| Statistics and Drawing | R | 4.1.2 | <https://www.r-project.org/> |
| Statistics and Drawing | python | 3.8.12 | <https://www.python.org/> |
| drawing | pheatmap | 1.0.12 | <https://cran.r-project.org/web/packages/pheatmap/index.html> |
| drawing | ggplot2 | 3.4.4 | <https://github.com/tidyverse/ggplot2> |
| drawing | matplotlib | 3.5.1 | <https://matplotlib.org/> |
| Evolutionary tree | ggtree | v3.10.0 | <https://www.bioconductor.org/packages/release/bioc/html/ggtree.html> |
| Difference analysis | metagenomeSeq | 1.36.0 | Paulson JN, Stine OC, Bravo HC, et al. Differential abundance analysis for microbial marker-gene surveys. Nat Methods. 2013; 10(12):1200-1202. <https://bioconductor.org/packages/release/bioc/html/metagenomeSeq.html> |
| Difference analysis | LEfSe | 1.1.2 | Segata N, Izard J, Waldron L, et al. Metagenomic biomarker discovery and explanation. Genome Biol. 2011; 12(6):R60. <https://github.com/SegataLab/lefse> |
| Association analysis | vegan | 2.6.4 | <https://cran.r-project.org/web/packages/vegan/index.html> |
| Statistical analysis | scipy | 1.8.0 | <https://scipy.org/> |
| Statistical analysis | sklearn | 0.24.1 | <https://scikit-learn.org/stable/index.html> |
| Functional prediction | PICRUSt2 | 2 | Douglas, G.M., Maffei, V.J., Zaneveld, J.R. et al. PICRUSt2 for prediction of metagenome functions. Nat Biotechnol 38, 685–688 (2020). <https://huttenhower.sph.harvard.edu/picrust/> |
| Functional prediction | FAPROTAX | 1.2.1 | Louca S, Parfrey LW, Doebeli M. Decoupling function and taxonomy in the global ocean microbiome. Science. 2016; 353(6305):1272-1277. <http://www.loucalab.com/archive/FAPROTAX/lib/php/index.php?section=Home> |
| Functional prediction | FUNGuild | 1.1 | Nguyen, Nhu H. et al. “FUNGuild: An open annotation tool for parsing fungal community datasets by ecological guild.” Fungal Ecology 20 (2016): 241-248. <http://www.funguild.org/> |
| Species Notes | silva | 138 | Quast C, Pruesse E, Yilmaz P, et al. The SILVA ribosomal RNA gene database project: improved data processing and web-based tools. Nucleic Acids Res. 2013; 41(Database issue):D590-D596. <https://www.arb-silva.de/> |
| Species Notes | UNITE | 8.2 | Abarenkov K, Nilsson RH, Larsson KH, et al. The UNITE database for molecular identification and taxonomic communication of fungi and other eukaryotes: sequences, taxa and classifications reconsidered. Nucleic Acids Res. 2024; 52(D1):D791-D797. <https://unite.ut.ee/> |
| Species Notes | NT | 2024.7 | <https://ftp.ncbi.nlm.nih.gov/blast/db/> |

**Supplementary table 1** software and databases were used in this study

| Sample | Raw Reads | Clean Reads | Mean_len(bp) | Mean_Q |
| --- | --- | --- | --- | --- |
| A1 | 89206 | 83455 | 1456 | 24.04 |
| A2 | 100207 | 93256 | 1456 | 24.07 |
| A3 | 94398 | 87374 | 1456 | 24.21 |
| A4 | 95341 | 88642 | 1454 | 24.27 |
| A5 | 98512 | 91042 | 1454 | 24.33 |
| A6 | 80114 | 74894 | 1457 | 24.28 |
| A7 | 68104 | 63427 | 1458 | 24.34 |
| A8 | 75439 | 69977 | 1457 | 24.25 |
| A9 | 69182 | 64309 | 1458 | 24.17 |
| A10 | 44794 | 41509 | 1455 | 24.25 |
| A11 | 45961 | 42386 | 1453 | 24.23 |
| A12 | 55896 | 51722 | 1455 | 24.43 |
| A13 | 59843 | 55530 | 1455 | 24.1 |
| A14 | 71680 | 66698 | 1455 | 24.08 |
| A15 | 73644 | 68636 | 1459 | 24.27 |
| A16 | 75568 | 70427 | 1459 | 24.24 |
| A17 | 99883 | 93069 | 1458 | 24.21 |
| A18 | 100065 | 92663 | 1458 | 24.19 |
| A19 | 99495 | 93078 | 1462 | 24.66 |
| A20 | 99678 | 92678 | 1456 | 24.22 |
| A21 | 99737 | 93795 | 1461 | 24.37 |
| A22 | 99750 | 93172 | 1459 | 24.2 |
| A23 | 100337 | 93762 | 1456 | 24.11 |
| A24 | 99658 | 92344 | 1462 | 24.53 |
| A25 | 99668 | 92811 | 1456 | 24.23 |
| A26 | 99828 | 93245 | 1456 | 24.18 |
| A27 | 100154 | 91982 | 1452 | 24.14 |
| A28 | 99681 | 92878 | 1457 | 24.22 |
| A29 | 100093 | 93288 | 1456 | 24.18 |
| A30 | 99576 | 92338 | 1454 | 24.29 |

**Supplementary table 2** Summary of sequencing data after barcode demultiplexing, quality filtering, and primer trimming

| Sample | input | mapped | filtered | rared |
| --- | --- | --- | --- | --- |
| A1 | 83455 | 82781 | 82622 | 41030 |
| A2 | 93256 | 92492 | 92337 | 41030 |
| A3 | 87374 | 86744 | 86610 | 41030 |
| A4 | 88642 | 87943 | 87783 | 41030 |
| A5 | 91042 | 90322 | 90153 | 41030 |
| A6 | 74894 | 74360 | 74192 | 41030 |
| A7 | 63427 | 62996 | 62861 | 41030 |
| A8 | 69977 | 69451 | 69311 | 41030 |
| A9 | 64309 | 63829 | 63693 | 41030 |
| A10 | 41509 | 41144 | 41030 | 41030 |
| A11 | 42386 | 41963 | 41824 | 41030 |
| A12 | 51722 | 51273 | 51122 | 41030 |
| A13 | 55530 | 55127 | 54992 | 41030 |
| A14 | 66698 | 66120 | 65996 | 41030 |
| A15 | 68636 | 68140 | 68019 | 41030 |
| A16 | 70427 | 69813 | 69663 | 41030 |
| A17 | 93069 | 92332 | 92155 | 41030 |
| A18 | 92663 | 92019 | 91873 | 41030 |
| A19 | 93078 | 92687 | 92568 | 41030 |
| A20 | 92678 | 92105 | 91971 | 41030 |
| A21 | 93795 | 93385 | 93252 | 41030 |
| A22 | 93172 | 92647 | 92517 | 41030 |
| A23 | 93762 | 92996 | 92823 | 41030 |
| A24 | 92344 | 91855 | 91715 | 41030 |
| A25 | 92811 | 91900 | 91727 | 41030 |
| A26 | 93245 | 92401 | 92251 | 41030 |
| A27 | 91982 | 91390 | 91280 | 41030 |
| A28 | 92878 | 92352 | 92199 | 41030 |
| A29 | 93288 | 92578 | 92436 | 41030 |
| A30 | 92338 | 91854 | 91713 | 41030 |

**Supplementary table 3** Summary of feature analysis and sequence counts at each stage per sample

| **S.NO** | **GENE** | **FORWARD PRIMER** | **REVERSE PRIMER** | **PRODUCT LENGTH** |
| --- | --- | --- | --- | --- |
| 1. | VEGF | ATTCAGCGGACTCACCAGC | AGAACCAACCTCCTCAAACCG | 157 |
| 2. | BCL-2 | AGTACCTGAACCGGCATCTG | GACCCAGAATCCACTCACACC | 134 |
| 3. | BAX | TGGATCCAAGACCAGGGTGG | CTTCCCCCATTCATCCCAGG | 200 |
| 4. | P53 | TTCATTGGGACCATCCTGGC | TCTTCGGAGAAGCGTGACAC | 107 |
| 5. | Ki-67 | GAGGCTGAGACATGGAGACATAAT | CTTGACCTTCCCCATCAGGGT | 176 |

**Supplementary table 4** Primers used in the real time PCR

**^*^*^Epithelial integrity-^*** *^0: Normal, 1: Mild surface irregularity, 2:Moderate epithelial disruption, 3:Severe erosion/loss^*

***^*Goblet cell preservation-^*** *^0: Normal,1: Mild reduction, 2: Moderate depletion, 3: Severe depletion^*

***^*Inflammatory Cell infiltration-^*** *^0: None, 1: Mild, 2: Moderate, 3: Marked^*

***^*Crypt architecture^****^-0: Normal, 1: Mild distortion, 2: Moderate shortening and disorganization, 3: Severe crypt damage^*

**Supplementary table 5 Histological scoring of intestinal tissue across experimental groups.**
Quantitative assessment of intestinal histopathology showing differences in tissue integrity and structural alterations across groups.
